# Identification of Candidate Transcriptional Regulators of Epidermal Transfer Cell Development in *Vicia faba* Cotyledons

**DOI:** 10.3389/fpls.2016.00717

**Published:** 2016-05-25

**Authors:** Kiruba S. Arun-Chinnappa, David W. McCurdy

**Affiliations:** Centre for Plant Science, School of Environmental and Life Sciences, The University of NewcastleCallaghan, NSW, Australia

**Keywords:** *Vicia faba*, transfer cell, wall ingrowth, *trans*-differentiation, transcription factors, RNA-Seq

## Abstract

Transfer cells (TCs) are anatomically-specialized cells formed at apoplasmic-symplasmic bottlenecks in nutrient transport pathways in plants. TCs form invaginated wall ingrowths which provide a scaffold to amplify plasma membrane surface area and thus increase the density of nutrient transporters required to achieve enhanced nutrient flow across these bottlenecks. Despite their importance to nutrient transport in plants, little is known of the transcriptional regulation of wall ingrowth formation. Here, we used RNA-Seq to identify transcription factors putatively involved in regulating epidermal TC development in cotyledons of *Vicia faba*. Comparing cotyledons cultured for 0, 3, 9, and 24 h to induce *trans*-differentiation of epidermal TCs identified 43 transcription factors that showed either epidermal-specific or epidermal–enhanced expression, and 10 that showed epidermal-specific down regulation. Members of the WRKY and ethylene-responsive families were prominent in the cohort of transcription factors showing epidermal-specific or epidermal–enhanced expression, consistent with the initiation of TC development often representing a response to stress. Members of the MYB family were also prominent in these categories, including orthologs of MYB genes involved in localized secondary wall deposition in *Arabidopsis thaliana*. Among the group of transcription factors showing down regulation were various homeobox genes and members of the MADs-box and zinc-finger families of poorly defined functions. Collectively, this study identified several transcription factors showing expression characteristics and orthologous functions that indicate likely participation in transcriptional regulation of epidermal TC development in *V. faba* cotyledons.

## Introduction

Transfer cells (TCs) commonly *trans*-differentiate from various differentiated cell types at anatomical locations where enhanced rates of nutrient transport are required for programmed development or in response to stress (Offler et al., 2003). The characteristic feature of TCs is their development of extensive and morphologically varied wall ingrowth networks which provide a scaffold to increase plasma membrane surface area to enable high densities of membrane transporters and thus accommodate increased demand for nutrient transport (Offler et al., 2003; McCurdy et al., 2008).

Wall ingrowth formation in TCs represents a novel example of wall deposition in plant cells (McCurdy et al., 2008; McCurdy, 2015), presumably involving unique sets of genes to both initiate and then orchestrate the cellular events required for this highly localized wall deposition event. Indeed, transcript profiling in endosperm TCs of barley (Thiel et al., 2008, 2012) and epidermal TCs of *Vicia faba* cotyledons (Dibley et al., 2009; Zhang et al., 2015b) established that wall ingrowth deposition involves differential expression of many hundreds of genes, presumably organized within transcriptional cascades to coordinate expression of the biosynthetic machinery required for wall ingrowth deposition and TC function (Arun-Chinnappa et al., 2013). These studies identified a role for auxin and ethylene signaling in inducing TC development, and details of the involvement of epidermal-specific ethylene and reactive oxygen species signaling pathways in epidermal TC induction have been elucidated (Zhou et al., 2010; Andriunas et al., 2011, 2012).

A major unresolved question in TC biology is the identity of transcription factors which respond to these inductive signals to coordinate the downstream expression of biosynthetic machinery required for wall ingrowth building. In maize, *ZmMRP-1*, a member of the MYB-Related family of transcription factors, has been identified as a key regulator of basal endosperm TC development (Gómez et al., 2009). *ZmMRP-1* is expressed specifically in endosperm TCs (Gómez et al., 2002) and regulates the expression of several maize TC-specific genes such as *MEG-1* (*Maternally Expressed Gene 1*; Gutierrez-Marcos et al., 2004), *BETL-1* and *BETL-2* (Gómez et al., 2002), and *TCRR-1* (*Transfer Cell Response Regulator 1*; Mũniz et al., 2006). When transformed into *Arabidopsis thaliana* (Arabidopsis) or *Nicotiana tabacum*, the *ZmMRP-1* promoter drives expression at tissue locations involved in active transport (Barrero et al., 2009). Thus, while *ZmMRP*-1 clearly plays a key role in regulating basal endosperm TC development, the genetic origin of these cells which develop in the triploid endosperm as part of a cell fate specification pathway (Thiel, 2014) is clearly different from most TCs which develop via *trans*-differentiation. Thus, transcriptional regulation of the *trans*-differentiation of TCs forming reticulate wall ingrowths may likely be different from that occurring in basal endosperm TCs.

Dibley et al. (2009) used cDNA-AFLP to survey differential expression across *trans*-differentiation of epidermal TCs in cultured cotyledons of *V. faba*. The cotyledon culture system provides a valuable experimental approach for transcriptional profiling of TC development, since the adaxial epidermal cells form an experimentally accessible and homogenous population of cells which *trans*-differentiate into functional TCs when isolated cotyledons are placed adaxial surface down on nutrient agar (Farley et al., 2000; Offler et al., 2003; Dibley et al., 2009; Zhou et al., 2010; Andriunas et al., 2012). Dibley et al. (2009) used this experimental system in combination with cDNA-Amplified Fragment Length Polymorphism (AFLP) analysis to identify extensive transcriptional changes occurring across the *trans*-differentiation of epidermal cells to become functional epidermal TCs. However, due to the technical limitations of cDNA-AFLP as a platform for high-throughput gene discovery, this study identified only a small cohort of the larger population of genes predicted to display changes in epidermal-specific temporal expression. Amongst this analysis, only a few transcription factors were identified as differentially expressed, and thus the depth of discovery of such genes putatively involved in regulating the *trans*-differentiation of TCs was limited.

The development of RNA-Seq for transcriptional profiling in species without a sequenced genome, with its deep sequencing capabilities to detect changes in transcript abundance with high sensitivity (Simon et al., 2009; Martin et al., 2013; Weber, 2015), provides an opportunity to re-visit transcript profiling using the *V. faba* cotyledon culture system. Zhang et al. (2015b) used this approach to identify genes putatively involved in the inductive signaling of wall ingrowth deposition and the biosynthetic genes presumably involved in this process, but did not report the identities of transcription factors expected to switch on expression of biosynthetic genes in response to these signaling pathways. In this current study we used RNA-Seq to identify at least 43 transcription factors which show differential up-regulated expression in adaxial epidermal cells of *V. faba* cotyledons undergoing *trans*-differentiation to form epidermal TCs, and 10 which are similarly down-regulated. Many of the transcription factors showing up-regulation belong to the WRKY and Ethylene Response Factor families, consistent with *trans*-differentiation of epidermal TCs representing a stress response involving ethylene (Zhou et al., 2010; Andriunas et al., 2012). Furthermore, several members of the MYB family of transcription factors were also identified as differentially up-regulated, in particular *VfMYB20*, the ortholog of which in Arabidopsis, *AtMYB20*, is a key target of the NAC-domain secondary wall master regulators *VNT6* and *VNT7* (Kubo et al., 2005; Zhong et al., 2010). This result implies possible similarity in the transcriptional pathways regulating secondary wall deposition and wall ingrowth formation in plants.

## Materials and methods

### Plant growth and cotyledon culture

*V. faba* L. (cv. Fiord) plants were grown in environmentally-controlled glasshouse and growth cabinet conditions as previously described (Offler et al., 1997; Farley et al., 2000). To sample transcripts from cotyledons undergoing epidermal TC development, cotyledons (100–120 mg FW) were removed from pods and either fixed immediately (*t* = 0-h) in ice-cold ethanol:acetic acid (3:1 v/v) for 1 h, or placed adaxial surface down on filter paper soaked in Murashige and Skoog medium (MS; Sigma Australia) in petri dishes. The Petri dishes were sealed with tape and incubated in darkness at 22°C for 3, 9, or 24 h, then the cotyledons were fixed in ice-cold ethanol:acetic acid as described above. The ethanol-acetic acid fixation was used for all cotyledons to minimize wounding responses associated with handling. Fixed cotyledons were rinsed briefly in distilled water and sheets of adaxial epidermal cells and discs of storage parenchyma were then isolated from each cotyledon as described (Dibley et al., 2009). For each time-point (0, 3, 9, 24 h) epidermal peels and storage parenchyma disks were collected from a minimum of 10 cotyledons derived from pods harvested from at least three different plants, and were snap frozen in liquid nitrogen and stored at –80°C prior to RNA isolation.

### Total RNA isolation and cDNA library preparation

Total RNA was extracted from the samples described above using an RNeasy Plant RNA isolation kit (Qiagen) incorporating on-column DNAse digestion. For RNA-Seq, between 1 and 2 μg of total RNA was isolated as described above from each sample and subjected to quality assurance (QA) analysis using an Agilent 2100 Bioanalyzer. One hundred-bp single-end read libraries were prepared for each sample using the Illumina TrySeq Library kit and sequencing was performed using the Illumina HiSeq-2000 platform. The QA analysis, library construction and 100-bp single-end sequencing were performed by the Australian Genome Research Facility (Melbourne).

### RNA-seq analysis

Sequencing of the poly(A)^+^ transcriptome of epidermal cells and storage parenchyma of cultured cotyledons yielded 368 M reads in total (42–48 M reads for each of the eight samples). Raw reads of all eight samples have been deposited in the Short Read Archive database (NCBI) under accession number SRP071162. Raw reads from each sample were trimmed to remove low quality bases and then mapped against a *de novo* assembled, genome-wide transcriptome map generated using CLC Genome Workbench (Arun-Chinnappa and McCurdy, 2015). To determine transcript abundance, Reads Per Kilobase per Million (RPKM) mapped read values were calculated to determine normalized gene expression of each transcript at each time point using default settings in CLC Genome Workbench. The RPKM values were exported into Microsoft® Excel® 2010 for all subsequent data calculations. To rationalize fold-change calculations, contigs with RPKM values less than 0.1 were assigned a value of 0.1, and a value of 0.5 or above for a given contig was deemed to be expressed (Sweetman et al., 2012).

Calculated RPKM values for both epidermal cells and storage parenchyma, at all four time points, were used for differential expression analysis. Transcripts with RPKM values less than 0.5 in all time points, and thus deemed not to be expressed, were removed from all data sets, and fold-change based on RPKM values at 3, 9, and 24 h relative to the 0-h control was calculated for all transcripts expressed in each tissue type. To identify transcripts showing epidermal-specific up-regulation, a two-step filtering process was undertaken. First, all transcripts expressed in epidermal cells showing a two-fold (Log_2_2 = 1) or greater increase across at least one culture time point relative to 0-h were selected. Next, transcripts from this list that showed a fold-change of 1.2 (Log_2_1.2 = 0.26) or greater change in expression in storage parenchyma tissue across any of the culture time points relative to 0-h were eliminated from this cohort. However, these threshold levels yielded an excessively high number of transcripts. Hence, to refine this list, the same two-step filtering process was undertaken but this time using fold-change of five (Log_2_5 = 2.3) or above to identify changes in epidermal gene expression, and then eliminate from this cohort transcripts that showed a fold-change of two (Log_2_2 = 1) or greater in storage parenchyma. This analysis identified a cohort of 444 transcripts that showed a five-fold or greater increase in expression in adaxial epidermal cells but two-fold or less change in storage parenchyma tissue across the three culture time points relative to 0-h control. A similar strategy was undertaken to identify transcripts which were down-regulated specifically in epidermal tissue in any of the three culture time points relative to 0-h, with this analysis identifying 172 transcripts categorized as epidermal-specific, down-regulated. For all transcripts excluded from this epidermal-specific, differential expression category (i.e., either up- or down-regulated specifically in epidermal tissue), fold-change in expression between 0− and 3-h culture was calculated for both epidermal and storage parenchyma data sets. The difference of fold-change for each transcript between epidermal and storage parenchyma was then calculated, and transcripts with a difference of 25 or greater were deemed to show “epidermal-enhanced, up-regulated” expression. This analysis identified 198 transcripts displaying epidermal-enhanced, up-regulated expression. Similar analysis of transcripts showing “epidermal-enhanced, down-regulated expression identified 13 transcripts in this category. The rationale for this analyses is described in Results.

### Reverse transcription-quantitative PCR (RT-qPCR)

Total RNA was extracted from both isolated epidermal peels and storage parenchyma disks of cotyledons cultured for 0− and 3-h as described above. For this analysis, epidermal peels and storage parenchyma were obtained from a minimum of 5-8 cotyledons from three replicate plants independently of those used in RNA-Seq experiments. cDNA was synthesized from 1 μg of total RNA from each sample using Superscript® III first strand synthesis system (Invitrogen) as per the manufacturer's instructions. PCR reactions were carried out using RotorGene SYBR Green PCR kit (Qiagen) as per manufacturer's instructions and using a RotorGene-Q (Qiagen) thermocycler. Cycling conditions were as follows: 95°C for 2 min followed by 40 cycles of 95°C for 10 s, 60°C for 30 s and 72°C for 30 s. Expression analysis was performed using the delta-delta method using Q-gene software (Simon, 2003) with *VfEF*α (*V. faba Elongation Factor alpha*, AJ222579) as the reference gene (Andriunas et al., 2012). Primer and gene details used for the RT-qPCR analysis are listed in Supplementary Table 4.

## Results

### RNA-seq analysis of adaxial epidermal TC development

Adaxial epidermal cells of cultured *V. faba* cotyledons *trans*-differentiate to become epidermal TCs but cells of the adjacent storage parenchyma tissue do not (Farley et al., 2000; Talbot et al., 2007; Dibley et al., 2009). Consequently, genes showing epidermal-specific change in expression upon cotyledon culture represent a pool of candidates likely to be involved in the *trans*-differentiation of epidermal TCs (Dibley et al., 2009; Zhang et al., 2015b). To identify transcription factors showing epidermal-specific up-regulation, we performed RNA-Seq of epidermal cells and storage parenchyma isolated from cultured cotyledons. An initial analysis of transcriptional changes using a two-fold or greater change in expression in epidermal cells across cotyledon culture compared to a 1.2-fold change in storage parenchyma yielded more than 2000 transcripts (data not shown). To provide a more stringent analysis of epidermal-specific expression we identified transcripts showing a five-fold (Log_2_5 = 2.3) or greater change in epidermal cells across any of the three culture time points (3−, 9−, 24-h) relative to 0-h cotyledon culture compared to a two-fold (Log_2_2 = 1) or less change in storage parenchyma. This analysis identified 444 transcripts (see Supplementary Table 1) that matched this definition for “epidermal-specific, up-regulation”. Of this collection, 22 were identified as transcription factors (Table 1) based on annotation against both Viridiplantae and Arabidopsis gene sets as determined previously (Arun-Chinnappa and McCurdy, 2015). From this list of transcription factors, 13 were up-regulated within the first 3-h of culture, while the remaining nine were up-regulated in epidermal cells by either 9- or 24-h of cotyledon culture (Table 1). The list of 23 transcription factors was headed by a GT-3B-like member of the trihelix gene family (Unigene 13378), which showed early and sustained epidermal-specific up-regulation across the 24-h culture period (Table 1). The remaining list of transcription factors shown in Table 1 is dominated by members of the MYB and MYB-like families (9/23) followed by ethylene responsive (3/23) and bHLH (3/23) transcription factors (Table 1).

**Table 1:** **Transcription factors displaying epidermal-specific, up-regulated expression in epidermal transfer cells of cultured *Vicia faba* cotyledons**.

**Unigene ID**	**Gene Product (Viridiplantae)**	**Species (Accession number)**	***E*-value (BLASTX)**	**Arabidopsis gene product (AT number)**	**Log_2_ Fold Change Epidermal and storage parenchyma (brackets) tissue compared to 0-h**		
					**3-h**	**9-h**	**24-h**
13378	Trihelix transcription factor GT-3B-like	*C. arietinum* (XP_004514215)	8e-49	Homeodomain-like superfamily protein (AT2G38250)	8.9 (−)	7.1 (−)	6.5 (−)
9668	P-type R_2_R_3_ MYB protein	*M. truncatula* (XP_003600714.1)	4e-115	ATMYB20 (AT1G66230)	7.1 (−)	5.6 (0)	4.9 (0.5)
7266	Ethylene-responsive transcription factor	*M. truncatula* (XP_003638785.1)	2e-97	ERF/AP2 transcription factor RAP2.4 (AT1G78080)	4.3 (0)	3.2 (0)	3.3 (0.4)
20101	Transcription factor bHLH122-like	*C. arietinum* (XP_004486946.1)	1e-97	Basic Helix-Loop-Helix (bHLH) (AT2G42280)	4.0 (−)	1.0 (−)	- (−)
19492	MYB family transcription factor-like protein	*M. truncatula* (XP_003618488.1)	4e-124	ATMYB30 (AT3G28910)	3.8 (0)	1.0 (–0.9)	0 (–1)
3890	R_2_R_3_-MYB transcription factor	*M. truncatula* (XP_003599668.1)	5e-152	ATMYB31 (AT1G74650)	3.7 (1)	2.3 (–0.2)	2.0 (0.4)
13430	DOF zinc finger protein	*M. truncatula* (XP_003638640.1)	1e-42	DOF zinc finger protein 1 (AT1G51700)	3.6 (0)	1.0 (0.4)	2.0 (0.8)
14644	Zinc finger (GATA type) family protein	*T. repens* (ADD09603.1)	6e-136	GATA transcription factor 5 (AT5G66320)	3.5 (0)	1.0 (0)	2.3 (–0.4)
12504	MYB transcription factor	*M. truncatula* (XP_003616176.1)	6e-144	MYB-like transcription factor (AT5G17300)	3.3 (–1)	2.0 (–0.6)	2.3 (–0.1)
20627	bHLH transcription factor	*M. truncatula* (XP_003626938.1)	6e-97	Basic Helix-Loop-Helix (bHLH) (AT3G07340)	3 (−)	1 (1.0)	1 (−)
16916	Transcription factor apetela 2	*M. truncatula* (XP_013465269.1)	9e-173	ERF/AP2 transcription factor RAP2.7 (AT2G28550)	3 (–2)	3 (–1)	1 (–2)
9906	Homeobox protein SBH1-like	*C. arietinum* (XP_004491125.1)	4e-147	KNOX/ELK homeobox transcription factor (AT1G62360)	2. (−)	1.0 (−)	1.0 (−)
7007	Ethylene-responsive transcription factor	*M. truncatula* (XP_003616666.1)	2e-56	ERF/AP2 transcription factor ABA INSENSITIVE 4 (AT2G40220)	2.6 (1)	1.0 (0.3)	1.0 (–0.1)
18946	TCP family transcription factor-like protein	*M. truncatula* (XP_003613691.1)	7e-103	TCP family transcription factor (AT1G69690)	2.3 (−)	1.0 (−)	1.0 (−)
771	Transcription factor bHLH78-like	*C. arietinum* (XP_004492265.1)	1e-173	Basic Helix-Loop-Helix (bHLH) (AT3G07340)	- (0)	2.8 (–0.5)	2.0 (0.2)
7111	MYB transcription factor	*M. truncatula* (XP_003624757.1)	9e-100	ATMYB60 (AT1G08810)	- (−)	4.4 (−)	3.8 (−)
17302	MYB transcription factor MYB56	*G. max* (NP_001235662.1)	2e-68	ATMYB4 (AT4G38620)	- (–1)	3.3 (–0.9)	3.3 (−)
19002	MYB-related protein MYB4-like	*C. arietinum* (XP_004488797.1)	4e-75	ATMYB63 (AT1G79180)	- (−)	3.6 (−)	5.0 (−)
12445	Transcription factor MYB113-like	*G. max* (XP_003529126.1)	3e-139	ATMYB114 (AT1G66380)	- (−)	0 (−)	2.8 (−)
13120	Transcription factor ASG4-like isoform X1	*C. arietinum* (XP_004497454.1)	2e-158	MYB-like transcription factor LHY-CCA1-LIKE5 (AT3G09600)	- (−)	0 (−)	2.3 (−)
10245	Ethylene-responsive transcription factor RAP2-6	*M. truncatula* (XP_003602747.1)	4e-99	ERF/AP2 transcription factor (AT5G61890)	-(−)	5.8 (−)	6.5 (−)
14898	WRKY transcription factor 28-like isoform X1	*C. arietinum* (XP_004491329.1)	3e-77	ATWRKY71 (AT1G29860)	0 (−)	1.0 (−)	4.7 (−)
21012	Ovate family protein 1	*M. truncatula* (AFK43560.1)	1e-25	Ovate family protein 1 (AT5G01840)	0 (0)	1.0 (0.9)	3.3 (0)

*Gene name and species correspond to annotation based on Viridiplantae data set. E-value refers to BLASTX comparison of the unigene sequence with the Viridiplantae gene. The orthologous gene from Arabidopsis and its AT number is listed. Log_2_ Fold Change was determined by calculating ratio of RPKM values at 3-, 9-, and 24-h culture compared to 0-h, for both epidermal (open numbers) and storage parenchyma (bracketed numbers). The term “−” indicates no expression, while the value “0” indicates fold change of 1, i.e., no difference in gene expression between the two time points*.

A similar strategy was undertaken to identify 172 transcripts that showed epidermal-specific down-regulation, defined as a five-fold or greater decrease in epidermal tissue with a two-fold or less change in storage parenchyma, across any culture time point relative to the 0-h control (see Supplementary Table 2). This list included 10 transcription factors, including two homeobox leucine zipper proteins, and transcription factors belonging to the MADS-box and zinc finger CCCH-domain families (Table 2).

**Table 2:** **Transcription factors displaying epidermal-specific, down-regulated expression in epidermal transfer cells of cultured *Vicia faba* cotyledons**.

**Unigene ID**	**Gene Product (Viridiplantae)**	**Species (Accession number)**	***E*-value (BLASTX)**	**Arabidopsis gene name (AT number)**	**Log_2_ Fold Change Epidermal and storage parenchyma (brackets) tissue compared to 0-h**		
					**3-h**	**9-h**	**24-h**
2074	Homeobox-leucine zipper protein ATHB-16	*M. truncatula* (XP_003613578.1)	1e-167	ATHB-1 (AT3G01470)	3.6 (–0.6)	0 (0.9)	0
1229	Homeobox-leucine zipper protein	*C. arietinum* (XP_004515372.1)	7e-113	Homeobox-leucine zipper protein 3 (AT3G60390)	3.5 (–0.2)	1.6 (0.2)	1.0 (0.3)
20713	MADS-box protein SOC1-like	*C. arietinum* (XP_004510894.1)	2e-84	AGAMOUS-like 20 (AT2G45660)	3.2 (−)	3.2 (−)	1.6 (−)
21180	Squamosa promoter-binding protein 1-like isoform X1	*G. max* (XP_003541730.1)	1e-48	Squamosa promoter binding protein-like 3 (AT2G33810)	2.8 (−)	3.3 (−)	3.3 (−)
7803	Zinc finger CCCH domain-containing protein 53-like	*C. arietinum* (XP_004513268.1)	0	Zinc finger (CCCH-type) family protein (AT3G51950)	2.6 (–0.2)	1.6 (0.6)	1.6 (–0.1)
16720	MADS-box transcription factor 12	*P. sativum* (AGV40795.1)	3e-139	MADS-box transcription factor family protein (AT4G18960)	2.3 (−)	2.8 (−)	1 (−)
14359	TMV resistance protein N	*M. truncatula* (XP_003612869.1)	5e-133	Disease resistance protein (TIR-NBS-LRR class) family (AT5G36930)	2.3 (−)	1.6 (−)	3.2 (−)
19507	mTERF domain-containing protein	*M. truncatula* (XP_003617710.1)	5e-157	Mitochondrial transcription termination factor family protein (AT5G55580)	1.6 (0.2)	2.6 (0.3)	1.0 (0)
464	AP2-like ethylene-responsive transcription factor ANT	*M. truncatula* (XP_003608564.1)	1e-75	AINTEGUMENTA (AT4G37750)	1 (0.8)	1.6 (1.8)	3.8 (0)
16491	High mobility group B protein 6-like	*C. arietinum* (XP_004503638.1)	6e-80	HMG (high mobility group) box protein (AT4G11080)	0 (–0.1)	1.0 (0)	2.8 (−0.4)

*Gene name and species correspond to annotation based on Viridiplantae data set. E-value refers to BLASTX comparison of the unigene sequence with the Viridiplantae gene. The orthologous gene from Arabidopsis and its AT number is listed. Log_2_ Fold Change was determined by calculating ratio of RPKM values at 0-h compared to 3-, 9-, and 24-h culture, for both epidermal (open numbers) and storage parenchyma (bracketed numbers). The term “-” indicates no expression, while the value “0” indicates fold change of 1, i.e., no difference in gene expression between the two time points*.

The criteria of using a five-fold or greater change to define a biologically relevant change in expression, and conversely a two-fold or less value to denote no change in expression, provides a stringent basis for claiming “epidermal-specific” change in expression, given the sensitivity of RNA-Seq to detect transcript abundance (e.g., Sweetman et al., 2012; Zhao et al., 2014). From this collective analysis, therefore, a total of 32 transcription factors were identified as showing epidermal-specific differential expression across adaxial epidermal TC development in *V. faba* cotyledons.

In addition to the genes listed in Table 1, we noted numerous examples in the RNA-Seq dataset where expression of a given transcript increased substantially in epidermal tissue within the first 3-h of cotyledon culture but its change in storage parenchyma was comparably less substantial but nonetheless greater than two-fold. To account for this cohort of genes excluded from the category of “epidermal-specific,” we calculated differences in fold-change between epidermal and storage parenchyma for each transcript at 3-h culture compared to 0-h control. Transcripts showing a numerical fold-change difference of 25 or greater were deemed as displaying “epidermal-enhanced, up-regulated” expression, whereby differential expression of these transcripts was 25 times greater in epidermal tissue compared to storage parenchyma. In total, 198 transcripts were identified in this category (see Supplementary Table 3), of which 20 were identified as putative transcription factors (Table 3). This cohort of transcription factors is characterized by high representation (nearly 50%) of members of the plant-specific WRKY family (Table 3), a result consistent with the *trans*-differentiation of epidermal TCs being a stress-related phenomenon regulated by ethylene (Andriunas et al., 2011, 2012). Similar analysis was carried out for transcripts excluded from Table 2 to identify down-regulated transcripts showing fold-change difference of 25 or greater between 0 and 3-h. A small cohort of 13 transcripts were identified as “epidermal-enhanced, down-regulated transcripts,” however no transcription factors were identified in this category (data not shown). These analyses were undertaken for early time points (0 and 3-h) only with the primary aim of identifying transcription factors regulating the *trans*-differentiation process.

**Table 3:** **Transcription factors displaying epidermal-enhanced, up-regulated expression in epidermal transfer cells of cultured *Vicia faba* cotyledons**.

**Unigene ID**	**Gene Product (Viridiplantae)**	**Species (Accession number)**	***E*-value (BLASTX)**	**Arabidopsis gene name (AT number)**	**Fold change**		**Fold difference**
					**EP**	**SP**	**EP fold change**
					**3-h**	**3-h**	***Minus***
					***v***	***v***	**SP fold change**
					**0-h**	**0-h**	
11848	WRKY transcription factor 28-like	*C. arietinum* (XP_004504707)	2e-71	ATWRKY28 (AT4G18170)	378	1	377
19283	Ethylene-responsive transcription factor	*M. truncatula* (XP_003630161.1)	1e-74	ABA REPRESSOR1 (AT5G64750)	332	7	325
10055	Transcription factor WRKY	*M. truncatula* (XP_003610463.1)	4e-142	ATWRKY6 (AT1G62300)	278	1	277
12104	WRKY transcription factor 33-like	*C. arietinum* (XP_004506827.1)	0	ATWRKY33 (AT2G38470)	191	1	190
7382	Hypothetical protein PHAVU_007G102900g	*P. vulgaris* (ESW15796.1)	5e-42	MYB-like transcription factor family protein (AT1G68670)	131	4	127
7356	Putative ethylene responsive factor	*V. faba* (ACD87816.1)	1e-50	ATERF98 (AT3G23230)	121	3	118
2088	WRKY transcription factor	*M. truncatula* (XP_003625994.)	5e-135	ATWRKY23 (AT2G47260)	81	2	79
10876	Ethylene-responsive transcription factor ERF110	*M. truncatula* (XP_003592075.1)	1e-61	ABA REPRESSOR1 (AT5G64750)	78	2	76
11312	WRKY transcription factor	*M. truncatula* (XP_003629812.1)	2e-137	ATWRKY65 (AT1G29280)	73	12	61
15437	Zinc finger and SCAN domain-containing protein	*M. truncatula* (XP_003604663.1)	1e-111	Homeodomain-like superfamily protein (AT2G38250)	69	1	68
3427	WRKY transcription factor 48-like	*C. arietinum* (XP_004486582.1)	3e-64	ATWRKY48 (AT5G49520)	64	1	63
20862	MYB family transcription factor APL	*M. truncatula* (XP_003613820.1)	1e-82	Homeodomain-like superfamily protein (AT1G69580)	48	2	46
5690	Transcriptional repressor NF-X1	*M. truncatula* (XP_003605239.1)	0	ATNFXL1 (AT1G10170)	46	2	44
2809	WRKY transcription factor WRKY100630	*M. truncatula* (ACD40316.1)	0	ATWRKY6 (AT1G62300)	46	2	44
10215	WRKY family transcription factor	*C. arietinum* (ABX10954.1)	5e-140	AtWRKY41 (AT4G11070)	42	2	40
18475	Transcription factor DIVARICATA-like isoform X1	*C. arietinum* (XP_004500230.1)	4e-69	Homeodomain-like transcriptional regulator (AT5G58900)	31	2	29
10039	Putative C_2_H_2_ type zinc finger protein	*M. truncatula* (XP_013457625.1)	2e-93	Unknown Protein (AT4G12450)	30	1	29
1309	NAC-domain protein	*M. truncatula*(XP_003602684.1)	2e-165	ANAC002 (AT1G01720)	29	2	27
19791	WRKY transcription factor 56	*M. sativa* (AEI83414.1)	2e-110	ATWRKY40 (AT1G80840)	27	2	25
18219	NAC domain- protein	*M. truncatula* (XP_003607287.1)	3e-142	ANAC036 (AT2G17040)	27	2	25

*Gene name and species correspond to annotation based on Viridiplantae data set. E value refers to BLASTX comparison of the unigene sequence with the Viridiplantae gene. The orthologous gene from Arabidopsis and its AT number is listed. “Fold Change” represents ratio derived for RPKM value from epidermal (EP) and storage parenchyma (SP) tissues at 3-h compared to 0-h culture. Fold difference is the difference between the fold change in epidermal tissue and storage parenchyma tissue. Only those transcripts showing a numerical fold-difference of ≥25 were selected, whereby differential expression of these transcripts in epidermal tissue is 25 times greater than in storage parenchyma*.

### Verification of expression by RT-quantitative PCR

**Figure 1:**
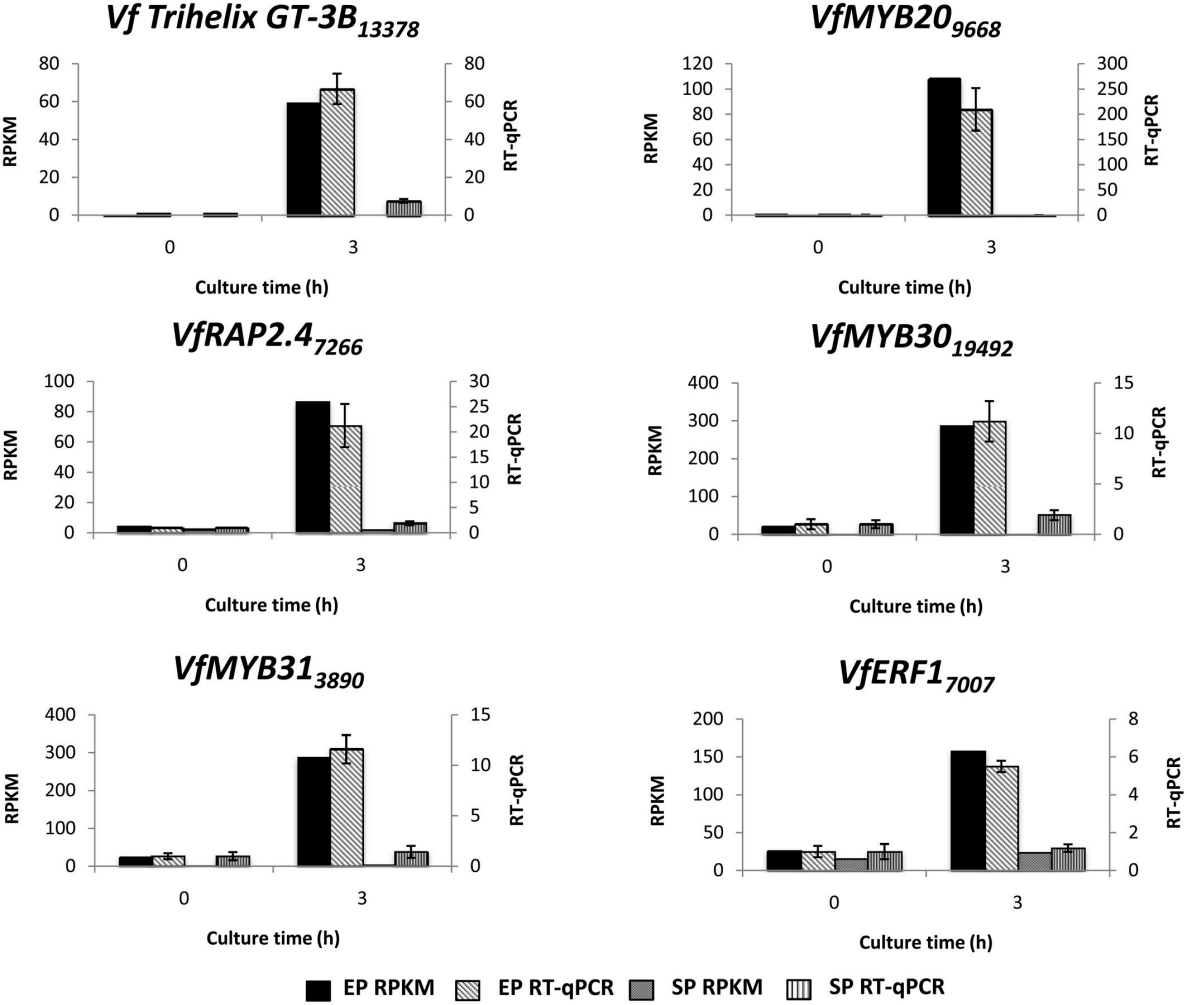
**Validation by RT-qPCR of selected transcription factor genes displaying epidermal-specific up-regulation as determined by RNA-Seq analysis**. Comparison of transcript expression determined by RNA-Seq (RPKM) and RT-qPCR across cotyledon culture (0- and 3-h) in both epidermal (EP) and storage parenchyma (SP) tissue. The RT-qPCR data is presented as expression normalized against *Vf Elongation Factor alpha* (Andriunas et al., 2012) at 0-h culture and is derived from the average of three biological replicates, with each replicate consisting of epidermal peels and storage parenchyma tissue obtained from 5-8 cotyledons (data shows mean ± SEM). Expression derived by RNA-Seq is indicated as RPKM. The subscript number associated with each *V. faba* transcript name indicates the relevant Unigene number.

**Figure 2:**
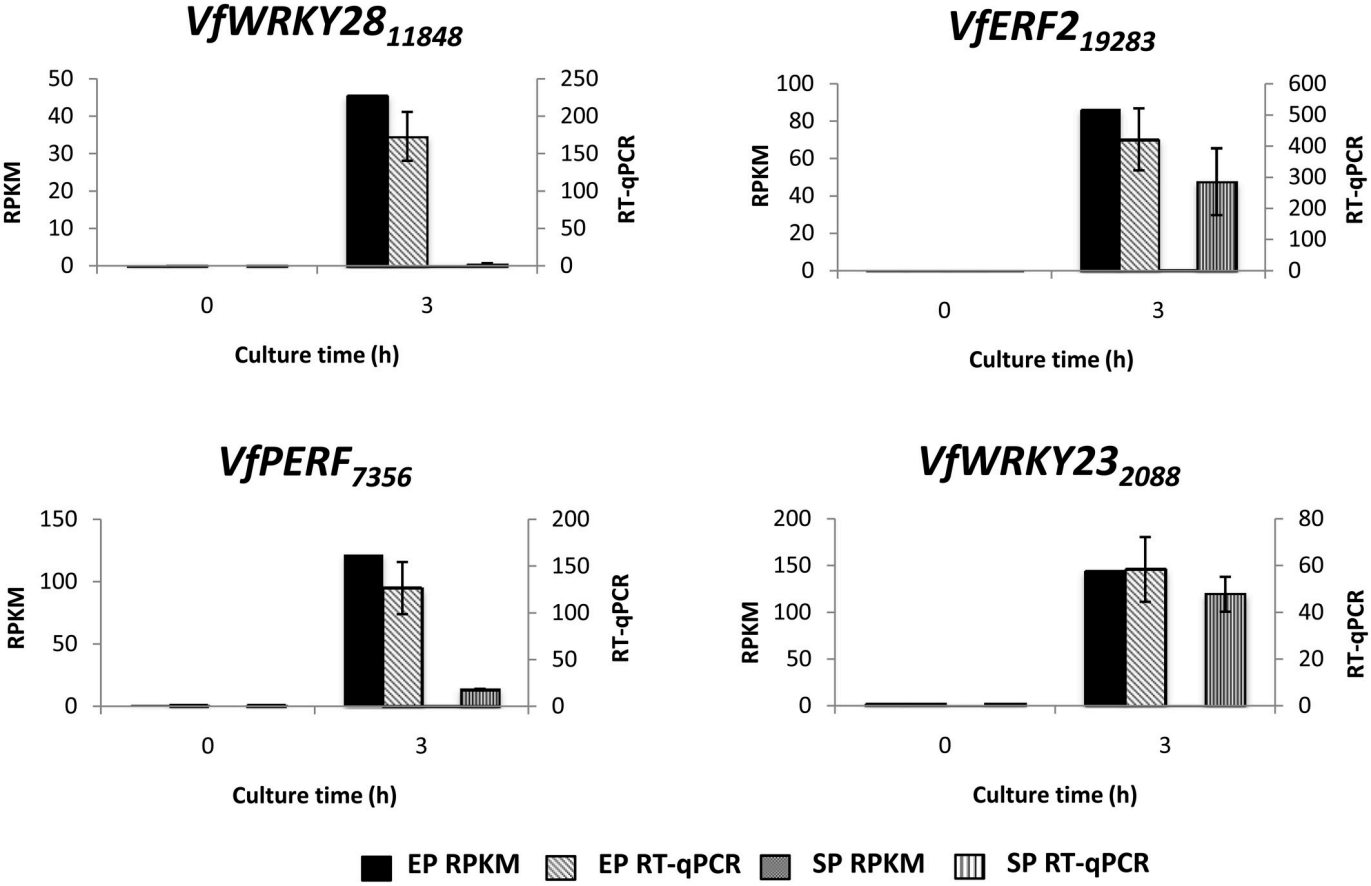
**Validation by RT-qPCR of selected transcription factor genes displaying epidermal-enhanced up-regulation as determined by RNA-Seq analysis**. Comparison of transcript expression determined by RNA-Seq (RPKM) and qRT-PCR across cotyledon culture (0− and 3-h) in both epidermal (EP) and storage parenchyma (SP) tissue. The RT-qPCR data is presented as expression normalized against *Vf Elongation Factor alpha* (Andriunas et al., 2012) at 0-h culture and is derived from the average of three biological replicates, with each replicate consisting of epidermal peels and storage parenchyma tissue obtained from 5-8 cotyledons (data shows mean ± SEM). Expression derived by RNA-Seq is indicated as RPKM. The subscript number associated with each *V. faba* transcript name indicates the relevant Unigene number.

To verify temporal expression of transcripts revealed by RNA-Seq, we performed RT-qPCR on a cohort of transcription factors showing substantial epidermal-specific (Table 1) or epidermal-enhanced (Table 3) up-regulated change in expression within 3-h of cotyledon culture. All six of the selected transcription factors (*VfTrihelix GT-3B, VfMYB20, VfRAP2.4, VfMYB30, VfMYB31, VfERF1*) showing epidermal-specific up-regulation as determined by RPKM showed expression essentially identical to or closely matching that obtained by RT-qPCR (Figure 1). This result therefore confirmed both the temporal (0 vs. 3-h) and spatial (epidermal vs. storage parenchyma) changes in expression for these genes across epidermal TC development. Of the four genes chosen as representative of epidermal-enhanced, up-regulated expression, RT-qPCR confirmed the expression profile of two, namely *VfWRKY28* and *VfERF*, whereas RT-qPCR recorded disparately high expression of *VfPERF2* and *VfWRKY23* in storage parenchyma tissue compared to RPKM values (Figure 2). The reason for this discrepancy for these two genes, and only in storage parenchyma tissue, is not known but may be due to non-specific amplification of a closely related sequence expressed predominantly in storage parenchyma. Irrespective of this possibility, in both cases RPKM values reported high differential expression of the relevant gene in epidermal tissue from 0− to 3-h compared to storage parenchyma (Figure 2). Collectively, the RT-qPCR analysis (Figures 1, 2) provide support for concluding that the RPKM values derived from the RNA-Seq analysis of cultured cotyledons accurately revealed temporal and spatial changes in expression across cotyledon culture, a conclusion supported by numerous studies using RNA-Seq for transcript profiling in non-sequenced plant species (e.g., O'Rourke et al., 2013; Liu et al., 2014).

## Discussion

In this study we used RNA-Seq to identify transcription factors putatively regulating the *trans*-differentiation of epidermal TCs in cultured *V. faba* cotyledons. Culturing *V. faba* cotyledons induces adaxial epidermal cells to form TCs, but cells of the neighboring storage parenchyma tissue do not, thus offering an experimental system to identify signaling pathways inducing epidermal TC *trans*-differentiation (Zhou et al., 2010; Andriunas et al., 2011, 2012) as well as global gene expression events associated with this process (Dibley et al., 2009; Zhang et al., 2015b). A similar study undertaken by Zhang et al. (2015b) was designed to identify genes involved in inductive signaling and subsequent building of the ingrowth wall network, but did not report on the identity of transcription factors presumably co-ordinating the expression of these biosynthetic genes. In contrast, the specific focus of this current study was to identify transcription factors potentially acting as transcriptional regulators of the *trans*-differentiation of epidermal TCs and thus linking inductive signaling with wall ingrowth building. Using cotyledon culture in combination with RNA-Seq, we identified 444 transcripts showing epidermal-specific differential expression, defined in this study as exhibiting a five-fold or greater temporal change in epidermal tissue across cotyledon culture, but only a two-fold or less change for the same transcript in storage parenchyma. These parameters were chosen to provide stringent criteria for classification as epidermal-specific differential expression, thus maximizing the potential to identify regulatory transcription factors based on substantive epidermal-specific change in expression, given that change in mRNA levels is the primary indicator for change in protein abundance in most instances (Weber, 2015). The total of 444 transcripts showing epidermal-specific differential regulation approaches the number of genes identified by cDNA-AFLP analysis of *V. faba* TCs (650 genes; Dibley et al., 2009) and microarray analysis of barley endosperm TCs (815 genes; Thiel et al., 2008). This correlation, however, is dependent on the criteria used to define epidermal-specific expression, and increased dramatically when a two-fold change was used in this instance (see Zhang et al., 2015b). Furthermore, excluded from our definition of epidermal-specific were a large number of transcripts showing substantial fold change in epidermal tissue and significantly less so in storage parenchyma, but nonetheless being two-fold or greater across cotyledon culture. This cohort was defined as showing epidermal-enhanced expression (Table 3, Supplementary Table 3), and as such contributed to the total number of genes displaying expression characteristics at least consistent with a role in regulating epidermal TC development.

A feature of the list of transcription factors showing epidermal-specific up-regulation is the prominence of members of the MYB gene family (Table 1). MYBs represent likely candidates to function as transcriptional regulators of wall ingrowth development in epidermal TCs since members of this family function as “second tier” transcriptional regulators of localized secondary wall deposition in plants (Zhong et al., 2010). In addition, the only transcription factor known to this point to regulate TC development, *ZmMRB1* from maize, is a member of the MYB-related family (Gómez et al., 2009). No *V. faba* ortholog of *ZmMRP1* was identified in the current study, but prominent among the list of MYBs was the *V. faba* ortholog of *AtMYB20* (Unigene 9668, Table 1), which showed a log_2_ fold change of 7.1 within 3 h of cotyledon culture. In Arabidopsis, *AtMYB20* sits downstream of *SND1* and *VND6/7*, both being NAC domain transcription factors identified as master switches for secondary wall deposition (Zhong et al., 2010). *AtMYB20* and its paralog *AtMYB43* are amongst a cohort of MYB factors activated by *SND1/VND6/7* and proposed to regulate transcriptional pathways responsible for deposition of cellulosic and hemicellulosic components of secondary walls (Nakano et al., 2010; Zhong et al., 2010). Interestingly, *V. faba* orthologues of *SND1/VND6/7* were not identified in our list of epidermal-specific, up-regulated transcripts, possibly due to rapid transient expression of these genes before the 3 h cotyledon culture time point, or alternatively that these secondary wall master switches do not participate in the regulation of *VfMYB20* in regulating TC development. At later stages of cotyledon culture, a *V. faba* ortholog of *AtMYB63* (Unigene 19002, Table 1) was also up-regulated specifically in epidermal cells. *AtMYB63* is a transcriptional activator of lignin biosynthesis required for secondary wall formation in Arabidopsis (Zhou et al., 2009; Zhong et al., 2010). The construction of secondary wall thickenings in xylem elements and wall ingrowths in TCs both represent examples of localized wall deposition in plants, thus the finding that *V. faba* orthologs of Arabidopsis genes known to be involved in secondary wall deposition are up-regulated specifically in epidermal cells upon wall ingrowth building, suggests overlapping roles in these processes. Contrary to this suggestion, however, is the observation that wall ingrowths do not contain lignin (Gunning and Pate, 1974; Vaughn et al., 2007), a circumstance consistent with the predicted diffusional properties of wall ingrowths (Gunning and Pate, 1974). However, Rocha et al. (2014) recently used TEM-energy dispersive X-ray spectrometry and acriflavine staining with confocal microscopy to demonstrate the presence of lignin in flange and so-called reticulate wall ingrowths in maize basal endosperm TCs. In this context, therefore, both the transcriptional data reported here and the findings of Rocha et al. (2014) suggest that the assumed absence of lignin in wall ingrowths may need to be revisited.

The transcription factor showing the highest fold-increase in epidermal cells upon cotyledon culture was an ortholog of the trihelix transcription factor GT-3B from chickpea (Unigene 13378, Table 1). The trihelix family of transcription factors contains 30 genes in Arabidopsis, but the majority have not been functionally defined (Ayadi et al., 2004; Kaplan-Levy et al., 2012). *AtGT-3B* is rapidly expressed in response to pathogen or salt stress (Park et al., 2004). In soybean, a similar response to these stresses is mediated by a calmodulin signaling gene, *SCaM-4*, which contains a GT-like element in its promoter recognized by *AtGT-3B* (Park et al., 2004). The function of the *V. faba* ortholog significantly up-regulated specifically in epidermal TCs is unclear, but wall ingrowth deposition in these cells involves calcium signaling (Zhang et al., 2015a), thus a *GT-3B-like* gene may be involved in this process.

Ethylene has been demonstrated to play a clear role in signaling the induction of epidermal TCs in *V. faba* cotyledons (Zhou et al., 2010; Andriunas et al., 2011). These TCs form by a *trans*-differentiation process which is preceded by dedifferentiation of the epidermal cells (Dibley et al., 2009). In this context it is noteworthy that a *V. faba* ortholog of the ERF/AP2 transcription factor *RAP2.4* (Unigene 7266, Table 1), now referred to as *WOUND INDUCED DEDIFFERENTIATION 1* (*WIND1*), was strongly induced in epidermal cells by 3-h of cotyledon culture. *WIND1* in Arabidopsis acts as a regulator of wound-induced dedifferentiation in young tissues, including cotyledon epidermal cells (Iwase et al., 2011). The culture-induced *trans*-differentiation of epidermal TCs represents a wound response in this tissue, this being an example of indirect *trans*-differentiation involving dedifferentiation without cell division (Nguyen and McCurdy, in press). Consequently, rapid epidermal-specific expression of a *V. faba* ortholog of *WIND1* to drive dedifferentiation as a first step of *trans*-differentiation is consistent with this proposition. Another ERF/AP2 ortholog (Unigene 10245, Table 1) was induced specifically in epidermal cells at 9 h culture. The ortholog of this gene, *ERF BUD ENHANCER* (*EBE*), functions in regulating cell proliferation and is highly expressed during S-phase of the cell cycle (Mehrnia et al., 2013), an observation consistent with the endopolyploid status of adaxial epidermal cells of *V. faba* cotyledons (Dibley et al., 2009) and up-regulation of a TCP ortholog involved in endoreduplication in *Arabidopsis* (Unigene 18946, Table 1). The delayed expression of *EBE* upon cotyledon culture, however, does not correlate with the temporal increase in mitotic index in these cells (Dibley et al., 2009; Zhang et al., 2015b), thus a definitive role for the *V. faba* ortholog of *EBE* is unclear. Finally, expression of the *V. faba* ortholog of *AtERF98* (Unigene 7356, Table 3) suggests a role for ascorbic acid in wall ingrowth deposition. Ascorbic acid, via its capacity for generating reactive oxygen species in the cell wall (Fry, 1992), can increase wall extensibility during growth (Schopfer, 2002). *AtERF98* plays an important role in ascorbic acid production (Zhang et al., 2012) and the substantial up-regulation of the *V. faba* ortholog in an epidermal-enhanced manner (Table 3) suggests it may have a role in increasing ascorbic acid production in adaxial epidermal cells to promote cell wall loosening required for wall ingrowth deposition.

A prominent feature of the list of genes showing epidermal-enhanced up-regulation is the abundance of WRKY transcription factors (Table 3). This gene family is commonly involved in regulating stress tolerance in plants (Rushton et al., 2010), and thus it is not surprising that many are up-regulated throughout the whole cotyledon upon transfer to culture conditions. However, since these *WRKY* genes show substantial fold-increase in epidermal expression compared to storage parenchyma, a role in stress-induced induction of epidermal TCs is reasonable to conclude. This conclusion is strengthened by considering the *V. faba* ortholog of *AtWRKY28* which shows a fold increase of 378 in epidermal cells by 3 h of culture (Unigene 11848, Table 3). In *Arabidopsis*, the paralog of *AtWRKY28, AtWRKY8*, is up-regulated in the tocopherol-deficient mutant *vte2* (*vitamin E 2*) when grown at low temperature (Maeda et al., 2014). The low temperature phenotype of *vte2* includes enhanced levels of delocalized wall ingrowth deposition in phloem parenchyma TCs (Maeda et al., 2006, 2008), an observation consistent with participation of both *AtWRKY8* and the *V. faba* ortholog in genetic pathways regulating wall ingrowth deposition. A role for WRKYs in signaling epidermal TC development is also suggested by the epidermal-enhanced up-regulation of an ortholog of *AtWRKY23* (Unigene 2088, Table 3). This gene in Arabidopsis is expressed during syncytia and giant cell formation in roots of nematode-infected cells (Grunewald et al., 2008; Cabrera et al., 2014), structures with distinct wall ingrowth-like characteristics. Furthermore, the Arabidopsis ortholog of Unigene 12104 (Table 3), *AtWRKY33*, regulates genes involved in the ethylene biosynthesis pathway (Birkenbihl et al., 2012), while the expression of both *AtWRKY23* and *AtWRKY33* is regulated by auxin (Berendzen et al., 2012; Grunewald et al., 2012). Hence, these two *WRKY* orthologs in *V. faba*, both of which show strong epidermal-enhanced expression (Table 3), are possibly induced by auxin and in turn act to promote ethylene biosynthesis as part of the auxin/ethylene signaling pathway which drives epidermal TC development in *V. faba* cotyledons (Zhou et al., 2010; Andriunas et al., 2011). Interestingly, of the *WRKY* genes appearing in Tables 1, 3, four of them (*AtWRKY23, 28, 71*, and *48*) cluster within the Ib subgroup of this family (Wu et al., 2005), suggesting structurally-conserved roles in signaling wall ingrowth deposition in TCs.

Homeobox transcription factors are prominent among the list of *V. faba* genes showing epidermal-specific down-regulated expression across cotyledon culture (Table 2). One of these, the Arabidopsis ortholog of Unigene 2074 (Table 2), *ATHB-1*, which belongs to the HD-Zip I class of transcription factors (Ariel et al., 2007), regulates genes involved in cell wall composition and cell elongation (Capella et al., 2015), and thus the downregulation of the *V. faba* ortholog may contribute to the altered compositional profile of wall ingrowths in epidermal TCs compared to the primary wall (Vaughn et al., 2007). A second *V. faba* homeobox transcription factor (Unigene 1229, Table 2), the Arabidopsis ortholog of which is *HAT3*, is a member of the HD-Zip II sub-family and participates in regulating apical embryo development and meristem function (Turchi et al., 2013). The functional significance in switching off expression of such a gene to involvement in the *trans*-differentiation of epidermal TCs is not immediately obvious, but this observation extends to many of the genes listed in Table 2. The *trans*-differentiation of epidermal TCs involves dedifferentiation followed by redifferentiation, processes that require substantial genomic reorganization and modifications (Dibley et al., 2009), thus switching off many of the genes listed in Table 2 may be a requirement to achieve these outcomes.

Collectively, this study has identified several transcription factors which due to their expression characteristics and orthologous functions in other systems represent likely candidates to participate in the transcriptional pathways leading to wall ingrowth deposition in epidermal TCs. The challenge now is to establish causal relationships between these genes and roles in regulating aspects of epidermal TC development in *V. faba* cotyledons. A robust transformation system for *V. faba* is not available, thus making a genetic approach to this challenge difficult. However, the induction of wall ingrowth deposition in epidermal TC of cultured cotyledons can be substantially delayed in the presence of inhibitors affecting auxin and ethylene signaling (Zhang et al., 2015a), thus making delivery of RNAi or other constructs by biolistic bombardment a possibility to demonstrate a role for these candidates in regulating epidermal TC development.

## Author contributions

DM designed the experiment; KA performed the experimental work; KA and DM analyzed the data; DM wrote the paper with assistance from KA.

## Funding

This project was supported by funds from an ARC Discovery Grant (DP110100770) and a University of Newcastle Faculty of Science and Information Technology Strategic Research Initiative Grant to DM. KA was supported by UNIPRS and UNRSC Scholarships.

### Conflict of interest statement

The authors declare that the research was conducted in the absence of any commercial or financial relationships that could be construed as a potential conflict of interest.
