# Supplementary material for: Identification of Candidate Transcriptional Regulators of Epidermal Transfer Cell Development in Vicia faba Cotyledons
Source: Front Plant Sci. 2016 May 25;7:717. doi: 10.3389/fpls.2016.00717 (PMC4879131; doi:10.3389/fpls.2016.00717)
Supplement: Supplementary file 4 [file Table4.DOCX]

**Identification of Candidate Transcriptional Regulators of Epidermal Transfer Cell Development in *Vicia faba* Cotyledons**

**Supplementary Table 4:** List of primers used for validation by qPCR analysis.

| **Unigene no.** | **Gene name** | **Primer sequences** |
| --- | --- | --- |
| 13378 | *Vf Trihelix GT-3B-like* | F:ATGGTGGTAGGTGGTGGAAG  R:TGCTTCCATTGTTTGTCTCC |
| 9668 | *VfMYB20* | F: CAACCATAACTGAAGAACATGACCA  R: CAACCATAACTGAAGAACATGACCA |
| 7266 | *VfRAP2.4* | F: ACAGGGAAAGCCAGAGAAGC  R: TCACTCTCAGTCATAGCCGGA |
| 19492 | *VfMYB30* | F:GTGTTTGTGTTTGTGATGTTGG  R:ACAGCTAGAGGACAATGGGAA |
| 3890 | *VfMYB31* | F:AGCAGCTCCATCATCAAAAAGC  R:CATGCATGCAAAAAGATCAACCAG |
| 7007 | *VfERF1* | F:ACGAGGAAGTAAAGGACGGA  R:CAACTGCTGAAGATGCTGCT |
| 11848 | *VfWRKY28* | F:ATGAAGACCCAACAACTGTGA  R:GGTGTTGTTATGATGGTGGTG |
| 19283 | *VfERF2* | F:TGGGCTGCTGAGATAAGAGA  R: CACTCTGAACCATTGGTGGAT |
| 7356 | *VfPERF* | F:ACTGCTGAACAAGCTGCAAG  R:ATGAAGAAGAAGGTGGATTAGGA |
| 2088 | *VfWRKY23* | F:CCACTAGCAGCTGAATGCAC  R:CATGATCCATAACCCTTGGA |

F: Forward primer; R: Reverse Primer
